# Supplementary material for: Satellite cell-derived TRIM28 is pivotal for mechanical load- and injury-induced myogenesis
Source: EMBO Rep. 2024 Aug 14;25(9):9. doi: 10.1038/s44319-024-00227-1 (PMC11387408; doi:10.1038/s44319-024-00227-1)
Supplement: Supplementary file 1 — Table EV1 [file 44319_2024_227_MOESM1_ESM.docx]

| **Antibodies** | **Source** | **Identifier** | **Dilution** |
| --- | --- | --- | --- |
| Anti-Mouse Total Tif1β (Kap-1, TRIM28) (C42G12) | Cell Signaling Technologies (Danvers, MA, USA) | Cat# 4124S | IHC: 1:30  WB: 1:1000 |
| Anti-HA-Tag (C26F4) | Cell Signaling Technologies (Danvers, MA, USA) | Cat# 3724S | IHC: 1:300 |
| Anti-Mouse P-Tif1β (KAP-1, TRIM28) Ser473 Poly6446 | BioLegend (San Diego, CA, USA) | Cat# 644602 | IHC: 1:30 |
| Anti-Mouse PAX7 | Developmental Studies Hybridoma Bank (Iowa City, IA, USA) | AB_528428 | IHC: 1:10 |
| Anti-Mouse MYH3 (embryonic myosin heavy chain) (F1.652) | Developmental Studies Hybridoma Bank (Iowa City, IA, USA) | AB_528358 | IHC: 1:350 |
| Anti-Mouse MYH2 (Type IIa myosin heavy chain) (SC-71) | Developmental Studies Hybridoma Bank (Iowa City, IA, USA) | AB_ 2147165 | IHC: 1:100 |
| Anti-Mouse MYH4 (Type IIb myosin heavy chain) (BF-F3) | Developmental Studies Hybridoma Bank (Iowa City, IA, USA) | AB_ 2266724 | IHC: 1:50 |
| Anti-Mouse MYH1 (Type IIx myosin heavy chain) (6H1) | Developmental Studies Hybridoma Bank (Iowa City, IA, USA) | AB_2314830 | IHC: 1:10 |
| Anti-Mouse MHC (all myosin heavy chain isoforms) (MF 20) | Developmental Studies Hybridoma Bank (Iowa City, IA, USA) | AB_2147781 | IHC: 1:50 |
| Anti-Mouse MYOD (5.8A) | BD Pharmingen (Franklin Lakes, NJ, USA) | Cat# 554130 | IHC: 1:50  WB: 1:1000 |
| Anti-Mouse Dystrophin (Dy8/6C5) | Novocastra (Leica Biosystems, Buffalo Grove, IL, USA) | Cat# NCL-DYS2 | IHC: 1:100 |
| Anti-Mouse MYOG (F5D) | Santa Cruz (Dallas, TX, USA) | Cat# sc-12732 | IHC: 1:50  WB: 1:500 |
| Anti-Mouse Myomixer | ThermoFisher Scientific (Waltham, MA, USA) | Cat# AF4580SP | WB: 1:2000 |
| Anti-Mouse Dystrophin | Abcam (Cambridge, UK) | Cat# ab15277 | IHC: 1:300 |
| Anti-Mouse Myomaker | Abcam (Cambridge, UK) | Cat# ab188300 | WB: 1:200 |
| Anti-Mouse Ki67 (SP6) | Abcam (Cambridge, UK) | Cat# ab16667 | IHC: 1:50 |
| Anti BrdU (BMC9318) | Sigma-Aldrich (St. Louis, MO, USA) | Cat# 11170376001 | IHC: 1:20 |
| Anti-Mouse Laminin | Sigma-Aldrich (St. Louis, MO, USA) | Cat# L9393 | IHC: 1:500 |
| Peroxidase-labeled Anti-Sheep Secondary | Sigma-Aldrich (St. Louis, MO, USA) | Cat# A3415 | WB: 1:1000 |
| Fab Fragment Goat Anti-Mouse IgG (H+L) Block | Jackson Immunoresearch (West Grove, PA, USA) | Cat# 115-007-003 | IHC: 1:10 |
| AMCA Anti-Mouse IgM | Jackson Immunoresearch (West Grove, PA, USA) | Cat# 115-155-075 | IHC: 1:150 |
| Alexa Fluor 488 Anti-Mouse IgG1 | Jackson Immunoresearch (West Grove, PA, USA) | Cat# 115-545-205 | IHC: 1:2000 |
| FITC Anti-Mouse IgG2b | Jackson Immunoresearch (West Grove, PA, USA) | Cat# 115-095-207 | IHC: 1:200 |
| Alexa Fluor 594 Anti-Mouse IgG1 | Jackson Immunoresearch (West Grove, PA, USA) | Cat# 115-585-205 | IHC: 1:2000 |
| Alexa Fluor 488 Anti-Rabbit IgG | Invitrogen (Carlsbad, CA, USA) | Cat# A11008 | IHC: 1:5000 |
| Alexa Fluor 594 Anti-Rabbit IgG | Invitrogen (Carlsbad, CA, USA) | Cat# A11037 | IHC: 1:5000 |
| Peroxidase-labeled Anti-Rabbit Secondary | Vector Labs (Burlingame, CA USA). | Cat# PI-1000 | WB: 1:5000 |
| Peroxidase-labeled Anti-Mouse Secondary | Vector Labs (Burlingame, CA USA). | Cat# PI-2000 | WB: 1:5000 |

Table EV1. List of antibodies used for immunohistochemical (IHC) and western blot (WB) analyses
