# Supplementary material for: Satellite cell-derived TRIM28 is pivotal for mechanical load- and injury-induced myogenesis
Source: EMBO Rep. 2024 Aug 14;25(9):9. doi: 10.1038/s44319-024-00227-1 (PMC11387408; doi:10.1038/s44319-024-00227-1)
Supplement: Supplementary file 2 — Appendix [file 44319_2024_227_MOESM2_ESM.pdf]

Appendix for

# Satellite cell-derived TRIM28 is pivotal for mechanical load- and injury-induced myogenesis

Kuan-Hung Lin and Jamie E. Hibbert, *et al.*

*Corresponding author. Email: troy.hornberger@wisc.edu

**Table of Contents**

Appendix Figure S1……………………………………………………………………3
Appendix Figure S2……………………………………………………………………4
Appendix Figure S3……………………………………………………………………5
Appendix Figure S4……………………………………………………………………6
Appendix Figure S5……………………………………………………………………7
Appendix Figure S6……………………………………………………………………8

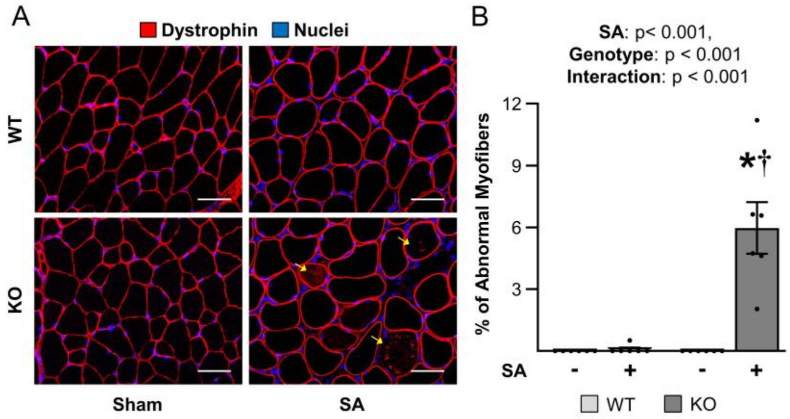


**Appendix Fig. S1. The loss of TRIM28 in satellite cells leads to the presence of myofibers with abnormal dystrophin at 14 days after the onset of synergist ablation.** Wild-type (WT) mice and tamoxifen-inducible satellite cell-specific TRIM28 knockout mice (KO) mice were treated with tamoxifen. At 14 days post tamoxifen, mice were subjected to unilateral synergist ablation surgery (SA+), with the non-ablated limb serving as a sham control (SA-). The mice were treated as described in Fig. 3 and the plantaris muscles were collected at 14 days after the SA surgery. (**A**) Mid-belly cross-sections were subjected to immunohistochemistry for dystrophin and nuclei. Yellow arrows indicate dystrophin detected within myofibers. (**B**) Quantification of myofibers that had an abnormal appearance of dystrophin. (two-way ANOVA, $n$ = 6/group, $p < 0.001$). Values are group means ± SEM. * indicates a significant effect of SA within the given genotype, † indicates a significant difference between the SA groups, $p < 0.05$. Scale bars = 50 µm.

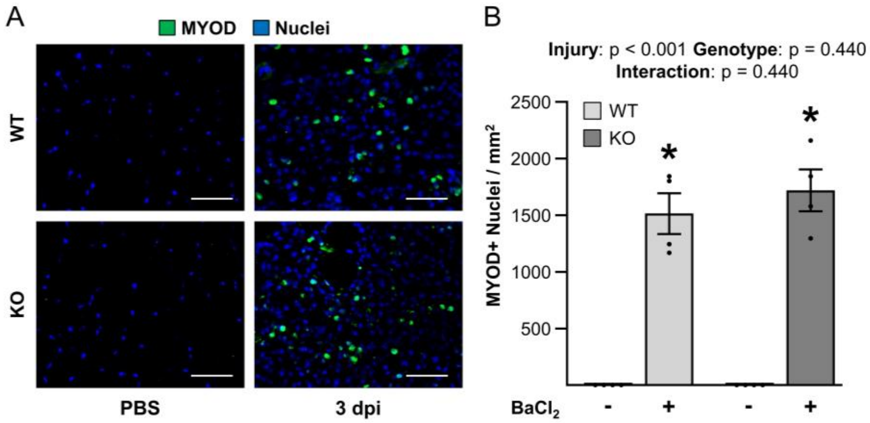


**Appendix Fig. S2. The loss of TRIM28 in satellite cells does not impair injury-induced satellite cell activation.** Wild-type (WT) mice and tamoxifen-inducible satellite cell-specific TRIM28 knockout mice (KO) mice were treated with tamoxifen. At 14 days post tamoxifen, their tibialis anterior muscles were injected with $BaCl_2$ (+) to induce injury or PBS (-) as a control condition. (**A**) The tibialis anterior muscles were collected at 3 days post-injury (dpi) and then mid-belly cross-sections were subjected to immunohistochemistry for MYOD (a marker of satellite cell activation), and nuclei. (**B**) Quantification of the number of MYOD-positive nuclei per $mm^2$ in A (two-way ANOVA, $n$ = 4/group, $p < 0.001$). Values are group means ± SEM. * indicates a significant effect of injury within a given genotype, $p < 0.05$. Scale bars = 50 µm.

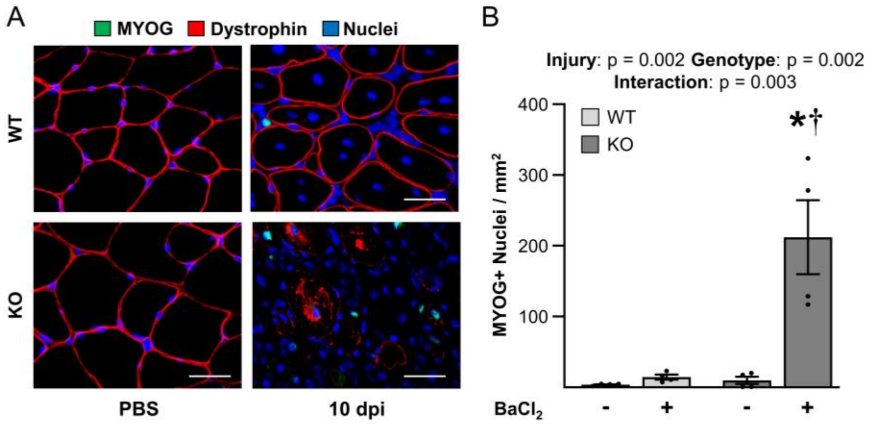


**Appendix Fig. S3. The loss of TRIM28 in satellite cells does not impair injury-induced satellite cell differentiation.** Wild-type (WT) mice and tamoxifen-inducible satellite cell-specific TRIM28 knockout mice (KO) mice were treated with tamoxifen. At 14 days post tamoxifen, their tibialis anterior muscles were injected with $BaCl_2$ (+) to induce injury or PBS (-) as a control condition. (**A**) The tibialis anterior muscles were collected at 10 days post-injury (dpi) and then mid-belly cross-sections were subjected to immunohistochemistry for MYOG, dystrophin, and nuclei. (**B**) Quantification of the number of MYOG-positive nuclei per $mm^2$ in A (two-way ANOVA, $n$ = 4/group, p = 0.002 for effect of both injury and genotype, p = 0.003 for interaction). Values are group means ± SEM. * indicates a significant effect of injury within a given genotype, † indicates a significant difference between the $BaCl_2$ treated groups, $p < 0.05$. Scale bars = 50 µm.

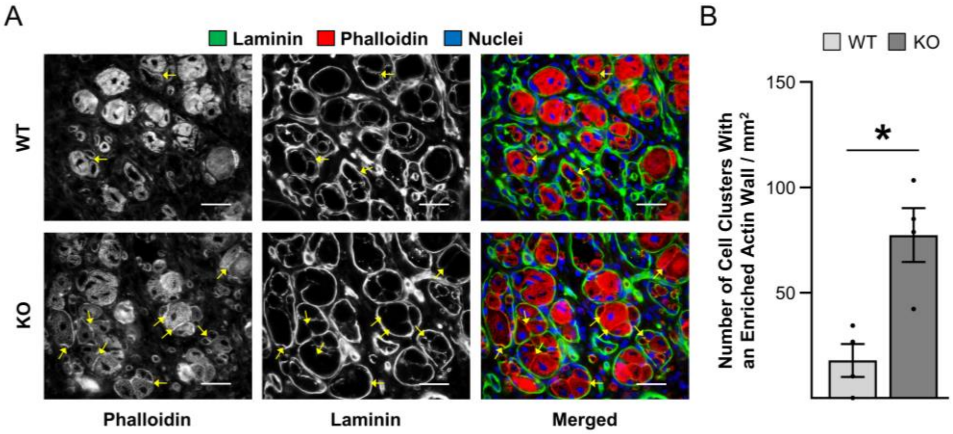


**Appendix Fig. S4. The loss of TRIM28 leads to an increase in the presence of dense walls of filamentous actin between well-aligned myoblasts/myofibers.** Wild-type (WT) mice and tamoxifen-inducible satellite cell-specific TRIM28 knockout mice (KO) mice were treated with tamoxifen. At 14 days post tamoxifen, their tibialis anterior (TA) muscles were injected with $BaCl_2$ to induce injury. (**A**) The tibialis anterior muscles were collected at 7 days post-injury and mid-belly cross-sections were subjected to immunohistochemistry for laminin, filamentous actin (i.e., phalloidin), and nuclei. (**B**) Clusters of well-aligned phalloidin-positive myoblasts/myofibers that were surrounded by a thick outer layer of laminin were identified, and then the number of clusters that contained phalloidin-positive myoblasts/myofibers with a dense wall of filamentous actin between the aligned cells (yellow arrows) was quantified (unpaired Student's t-test, $n = 4$/group, $p = 0.007385781$). Values are group means ± SEM. * indicates a significant difference between groups. $p < 0.05$. Scale bars = 50 μm.

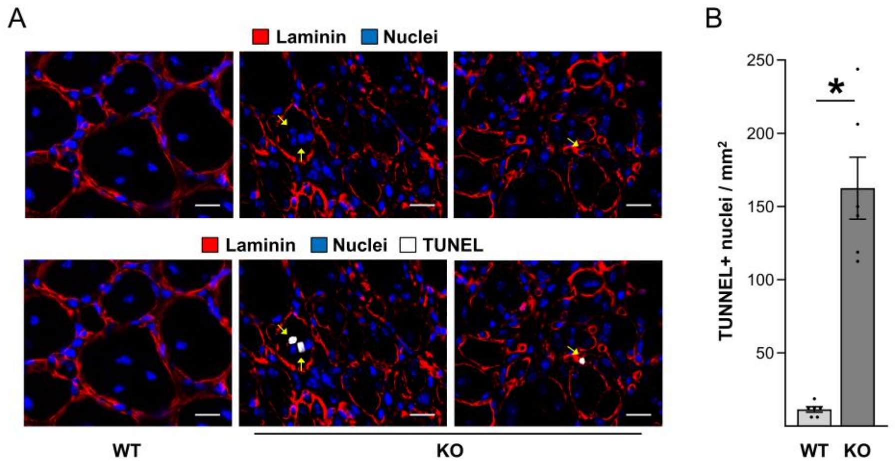


**Appendix Fig. S5. The loss of TRIM28 leads to an increase in the presence of TUNEL-positive nuclei.** Wild-type (WT) mice and tamoxifen-inducible satellite cell-specific TRIM28 knockout mice (KO) mice were treated with tamoxifen. At 14 days post tamoxifen, their tibialis anterior (TA) muscles were injected with $BaCl_2$ to induce injury. (**A**) The tibialis anterior muscles were collected at 10 days post-injury and mid-belly cross-sections were subjected to a TUNEL assay to detect fragmented DNA as a marker of apoptosis. The sections were then subjected to immunohistochemistry for laminin and nuclei. (**B**) The number of nuclei in each field that were TUNEL-positive (yellow arrows) was quantified (unpaired Student's t-test, $n$ = 6/group, p = 0.0000327). Values are group means ± SEM. * indicates a significant difference between groups. $p < 0.05$. Scale bars = 10 µm.

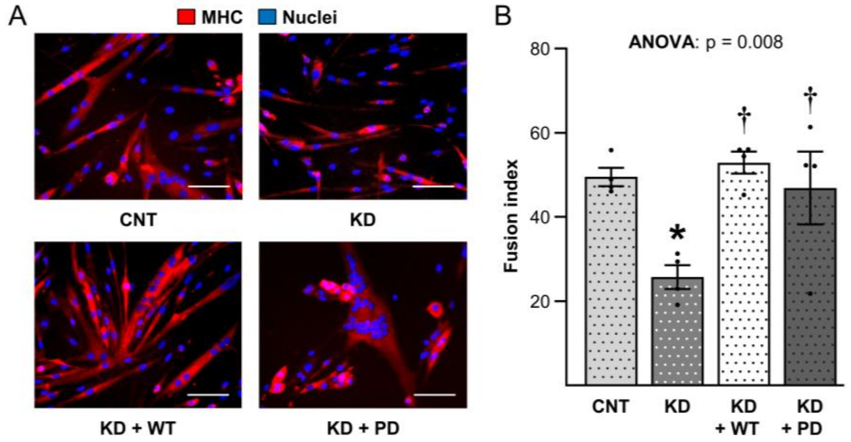


**Appendix Fig. S6. TRIM28(S473) phosphorylation is not required for fusion during *in vitro* myotube formation.** Primary myoblasts were infected with lentivirus encoding scrambled shRNA (CNT), shRNA targeting *Trim28* mRNA (KD), shRNA targeting *Trim28* mRNA along with "rescue" expression of a shRNA resistant form of TRIM28 (KD + WT), or shRNA targeting *Trim28* mRNA along with "rescue" expression of an shRNA resistant and phosphodefective form of TRIM28 (KD + PD) in which the serine 473 residue had been mutated to a non-phosphorylatable alanine. (**A**) Infected myoblasts were subjected to a myotube formation assay and immunohistochemistry for MHC and nuclei. (**B**) Quantification of the fusion index (% of nuclei inside MHC positive and multinucleated cells) (one-way ANOVA, $n = 4$/group with each sample representing an independent line of isolated primary myoblasts, $p = 0.008$). Values are group means ± SEM. * indicates a significant difference from CNT, † indicates a significant difference from KD, $p < 0.05$. Scale bars = 50 μm.
