# Supplementary figures and images for: Satellite cell-derived TRIM28 is pivotal for mechanical load- and injury-induced myogenesis
Source: EMBO Rep. 2024 Aug 14;25(9):9. doi: 10.1038/s44319-024-00227-1 (PMC11387408; doi:10.1038/s44319-024-00227-1)

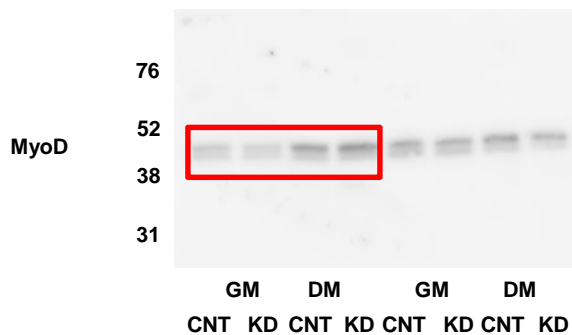

13% gel, cut between 17 and 24 kD markers.

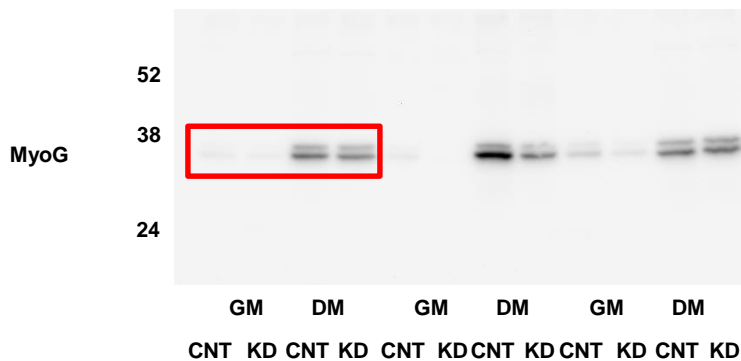

7.5% gel, cut at 76 kD markers.

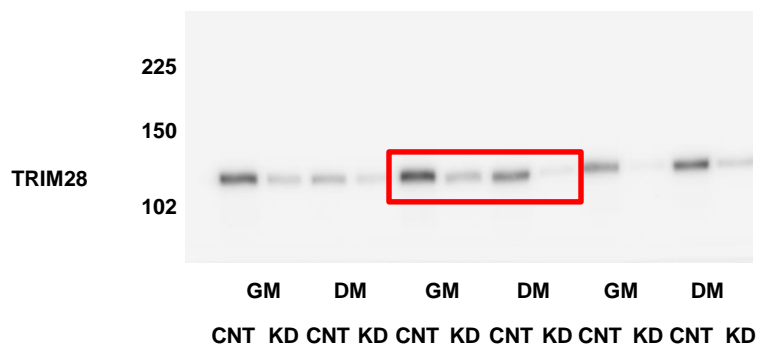

7.5% gel, cut between 76 kD markers.

Supplement: Supplementary file 9 — Source data Fig. 6 [file 44319_2024_227_MOESM9_ESM.zip › EMBOR-2024-58743V3_SourceDataForFig6/Fig. 6A.pdf]
