# Supplementary material for: Satellite cell-derived TRIM28 is pivotal for mechanical load- and injury-induced myogenesis
Source: EMBO Rep. 2024 Aug 14;25(9):9. doi: 10.1038/s44319-024-00227-1 (PMC11387408; doi:10.1038/s44319-024-00227-1)
Supplement: Supplementary file 13 — Source data Fig. 10 [file 44319_2024_227_MOESM13_ESM.zip › EMBOR-2024-58743V3_SourceDataForFig10/Fig. 10A.pdf]

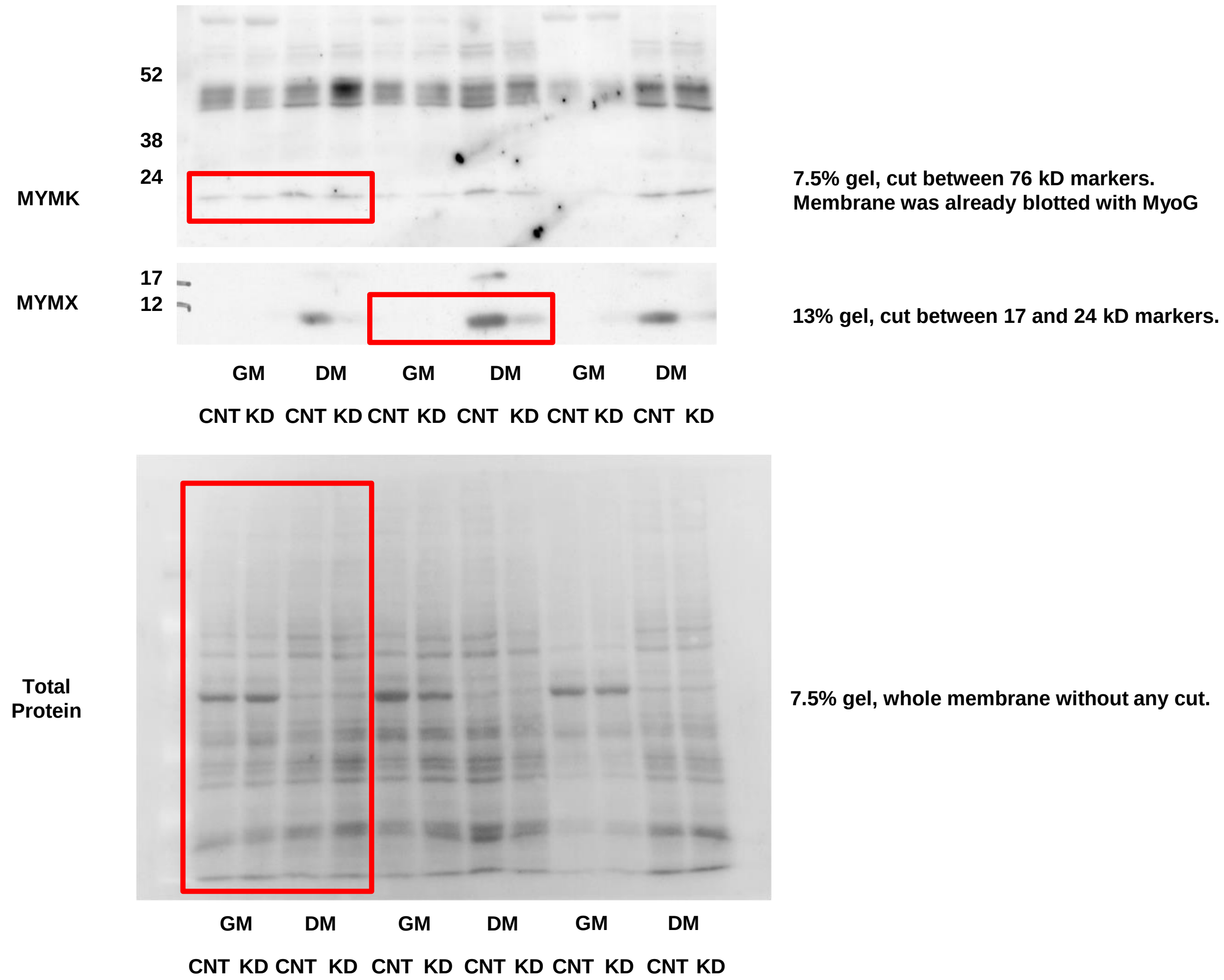
52
38
24
MYMK
7.5% gel, cut between 76 kD markers.
Membrane was already blotted with MyoG
17
12
MYMX
13% gel, cut between 17 and 24 kD markers.
GM DM GM DM GM DM
CNT KD CNT KD CNT KD CNT KD CNT KD CNT KD
Total Protein
7.5% gel, whole membrane without any cut.
GM DM GM DM GM DM
CNT KD CNT KD CNT KD CNT KD CNT KD CNT KD
