# Supplementary material for: Satellite cell-derived TRIM28 is pivotal for mechanical load- and injury-induced myogenesis
Source: EMBO Rep. 2024 Aug 14;25(9):9. doi: 10.1038/s44319-024-00227-1 (PMC11387408; doi:10.1038/s44319-024-00227-1)
Supplement: Supplementary file 14 — Expanded View Figures [file 44319_2024_227_MOESM14_ESM.pdf]

Expanded View Figures

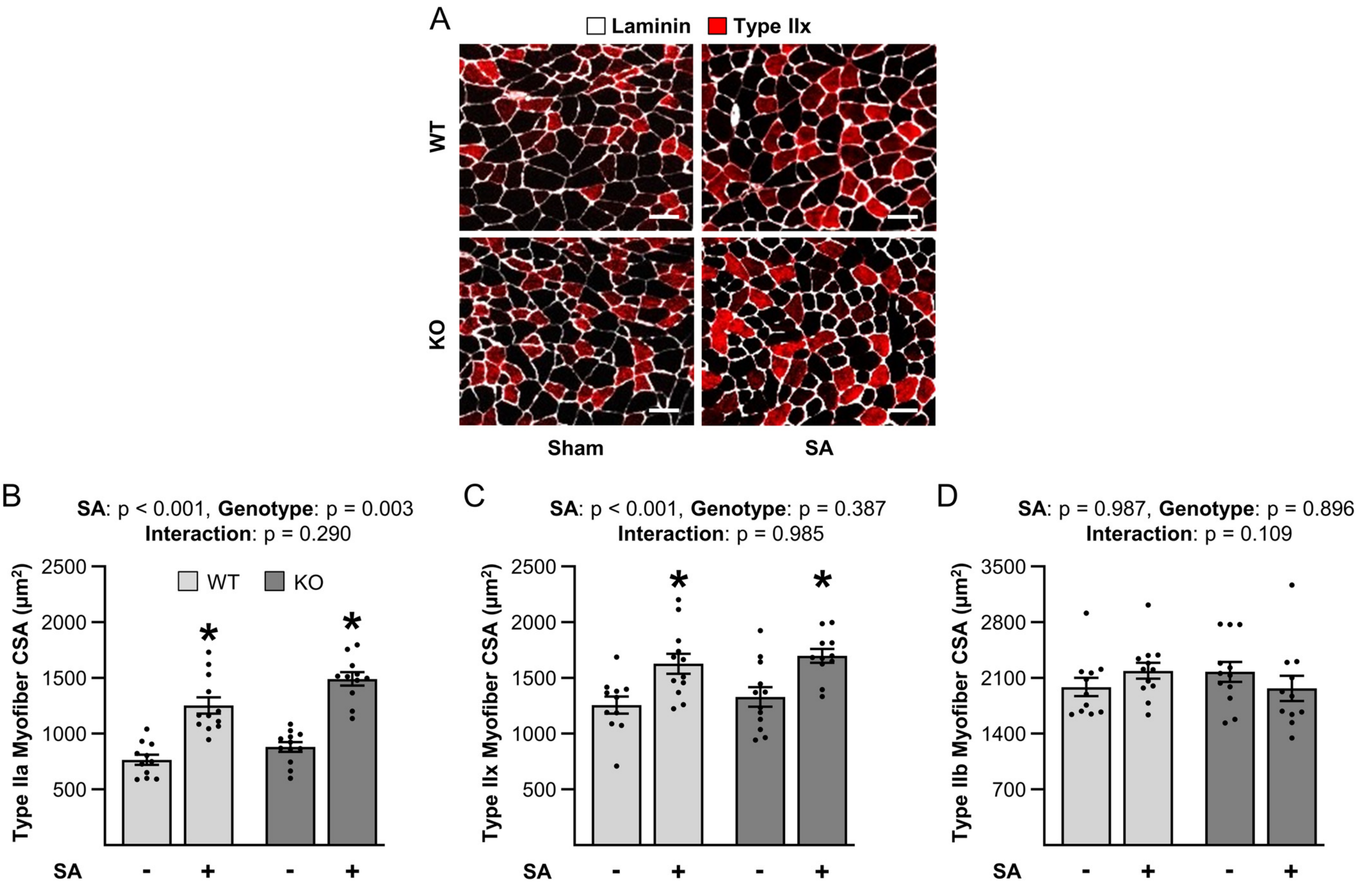

Figure EV1. The loss of TRIM28 in satellite cells does not impact the mechanical load-induced increase in the size of Type IIa or IIx myofibers.

Wild-type (WT) mice and tamoxifen-inducible satellite cell-specific TRIM28 knockout mice (KO) mice were treated with tamoxifen. At 14 days post tamoxifen, mice were subjected to unilateral synergist ablation surgery (SA+), with the non-ablated limb serving as a sham control (SA-). The mice were treated as described in Fig. 3 and the plantaris muscles were collected at 14 days after the SA surgery. (**A**) Mid-belly cross-sections were subjected to immunohistochemistry for laminin and type IIx myofibers. (**B–D**) Quantification of the type IIa (two-way ANOVA, $n = 11–12$/group, $^*p < 0.001$), type IIx (two-way ANOVA, $n = 11–12$/group, $^*p = 0.023$ or 0.0025), and type IIb (two-way ANOVA, $n = 11–12$/group) myofiber cross-sectional area, respectively. Values are group means + SEM. * indicates a significant effect of SA within the given genotype, $p < 0.05$. Scale bars = 50 µm.

© The Author(s)

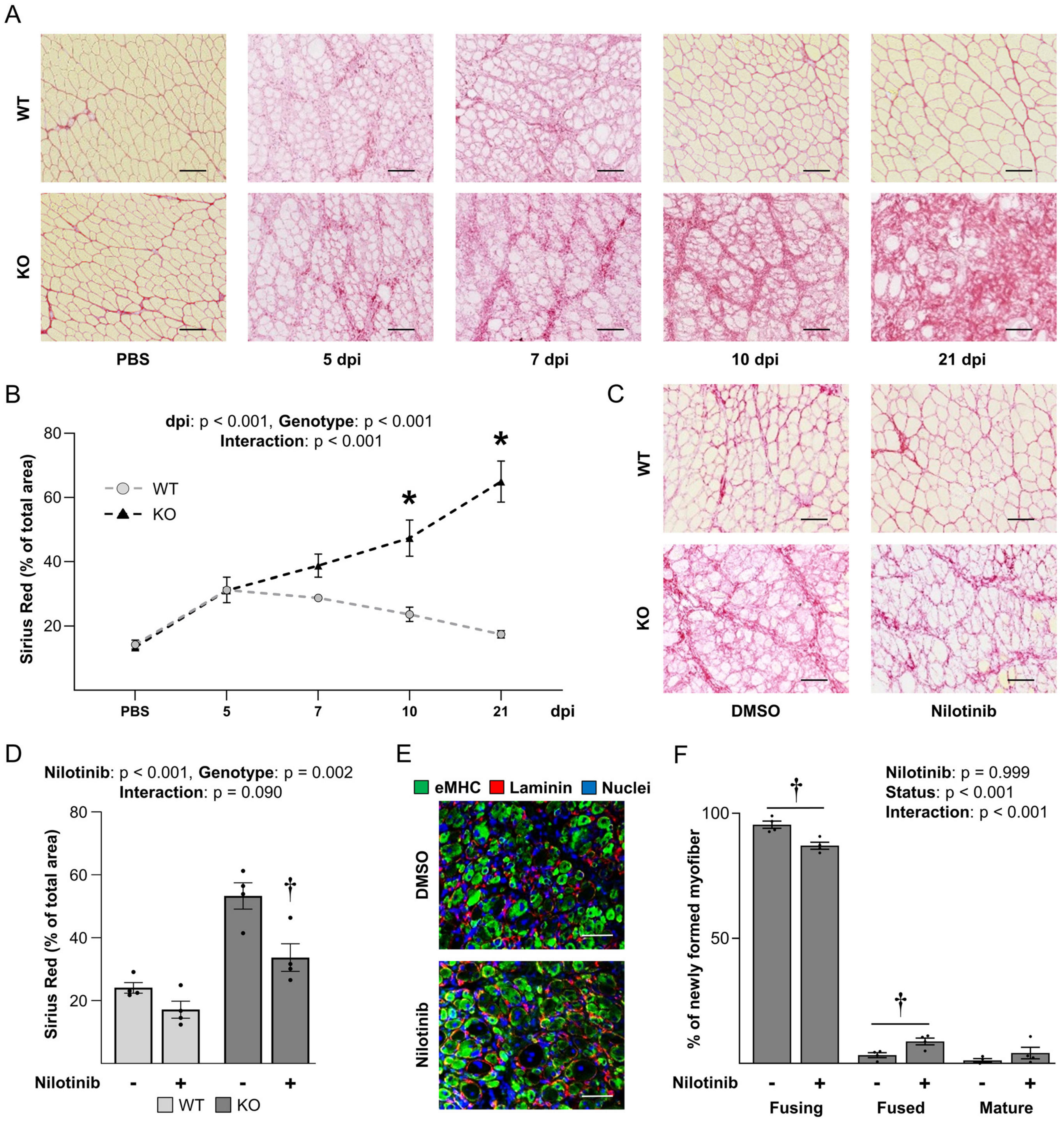
A
WT
KO
PBS
5 dpi
7 dpi
10 dpi
21 dpi
B
dpi: p < 0.001, Genotype: p < 0.001
Interaction: p < 0.001
WT
KO
Sirius Red (% of total area)
80
60
40
20
PBS
5
7
10
21
dpi
*
*
C
WT
KO
DMSO
Nilotinib
D
Nilotinib: p < 0.001, Genotype: p = 0.002
Interaction: p = 0.090
Sirius Red (% of total area)
80
60
40
20
Nilotinib
-
+
-
+
WT
KO
†
E
eMHC
Laminin
Nuclei
DMSO
Nilotinib
F
Nilotinib: p = 0.999
Status: p < 0.001
Interaction: p < 0.001
% of newly formed myofiber
100
50
Nilotinib
-
+
-
+
-
+
Fusing
Fused
Mature
†
†

 © The Author(s)

Figure EV2. The loss of TRIM28 in satellite cells leads to excessive fibrosis following BaCl2-induced injury, but reducing the fibrosis only minimally improves the impairment in fusion.

Wild-type (WT) mice and tamoxifen-inducible satellite cell-specific TRIM28 knockout mice (KO) mice were treated with tamoxifen. At 14 days post tamoxifen, their tibialis anterior (TA) muscles were injected with BaCl2 to induce injury or PBS as a control condition. (**A**) At 5, 7, 10, and 21 days post-injury (dpi), TA muscles were collected and mid-belly cross-sections were subjected to Sirius Red staining as a marker of collagen deposition. (**B**) Proportion of the muscle cross-section that stained positive for Sirius Red in (**A**) (two-way ANOVA, $n = 4$–6/group, *$p = 0.0001$ or <0.0001). (**C**–**F**) Daily intraperitoneal injections of Nilotinib (20 mg/kg/day) or DMSO were administered at 3 to 7 dpi. (**C**) TA muscles were collected at 10 dpi and mid-belly cross-sections were subjected to Sirius Red staining. (**D**) Proportion of the muscle cross-sections that stained positive for Sirius Red in (**C**) (two-way ANOVA, $n = 4$/group, *$p < 0.0001$, †$p = 0.0016$). (**E**) Mid-belly cross-sections of the TA muscles at 10 dpi were subjected to immunohistochemistry for eMHC, laminin, and nuclei. (**F**) Proportion of myoblasts/myofibers that were at the stage of fusing, fused, or mature as described in Fig. 4 (two-way ANOVA, $n = 4$/group, †$p = 0.0006$ or 0.0145). Values are group means + SEM. * indicates a significant difference between genotypes at the given condition, † significant effect of Nilotinib within the given genotype or stage $p < 0.05$. Scale bars = 100 µm in (**A**) and (**C**), and 50 µm in (**E**).

© The Author(s)

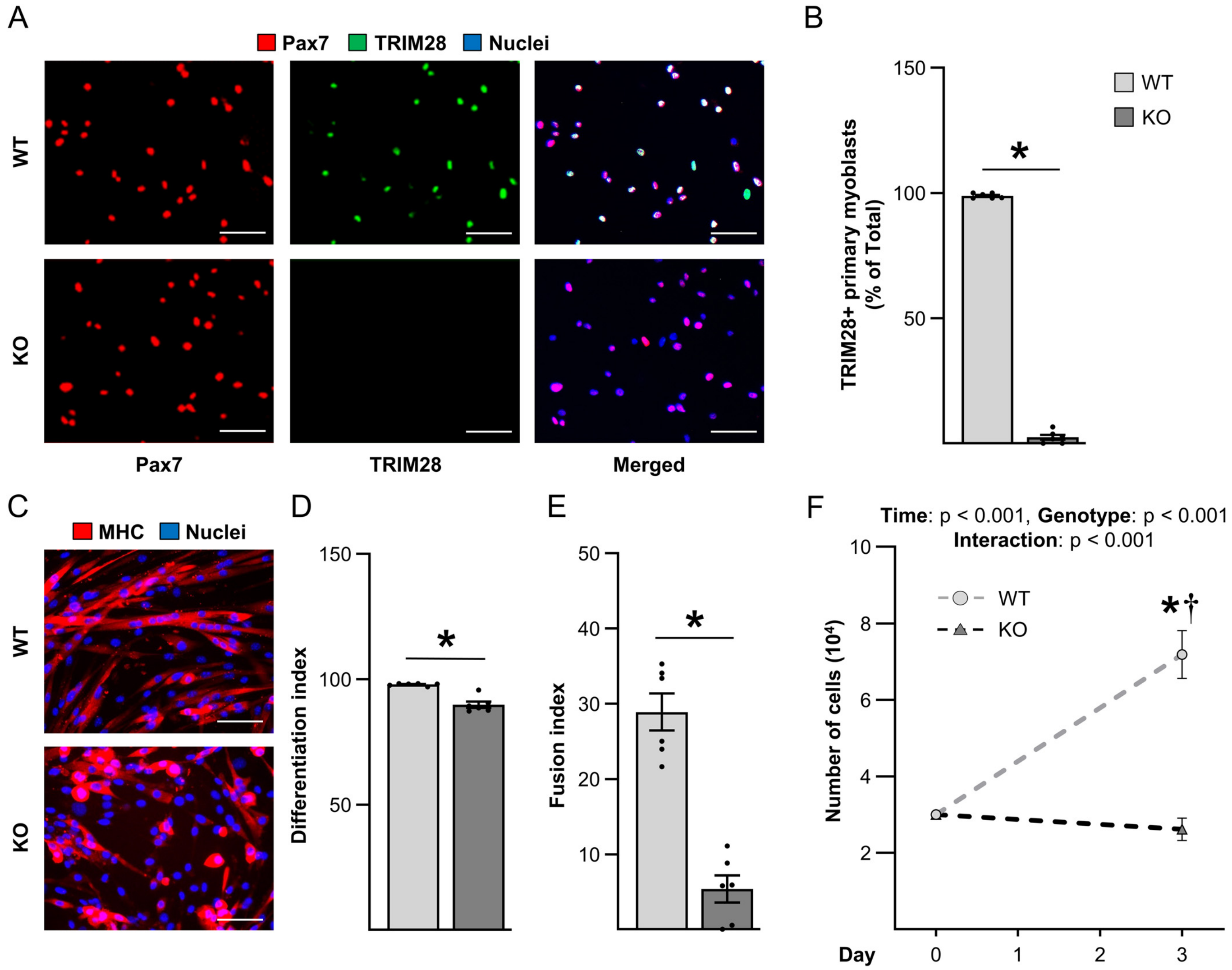

Figure EV3. The loss of TRIM28 in satellite cells leads to a fusion defect during in vitro myotube formation.

Wild-type (WT) mice and tamoxifen-inducible satellite cell-specific TRIM28 knockout mice (KO) mice were treated with tamoxifen. (**A**) At 14 days post tamoxifen, primary myoblasts were isolated, cultured in growth medium, and then subjected to immunohistochemistry for Pax7, TRIM28, and nuclei. (**B**) The proportion of the Pax7 positive primary myoblasts that expressed TRIM28 in (**A**) (unpaired Student's t-test, $n = 6$/group, *$p < 0.0001$). (**C–E**) WT and KO primary myoblasts were subjected to a myotube formation assay and immunohistochemistry for myosin heavy chain (MHC) and nuclei. (**D**) The differentiation index (% of nuclei inside MHC positive cells) (unpaired Student's t-test, $n = 6$/group, *$p < 0.0001$), and (**E**) the fusion index (% of nuclei inside MHC positive multinucleated cells) (unpaired Student's t-test, $n = 6$/group, *$p < 0.0001$) were quantified. (**F**) 3×10^4 primary myoblasts were seeded on day 0 and cultured in growth medium. The number of primary myoblasts was quantified on day 3 (two-way repeated-measures ANOVA, $n = 6$/group, * and † $p < 0.0001$). Values are group means + SEM, each sample representing an independent line of isolated primary myoblasts. * indicates a significant difference between genotypes within a given condition, † indicates a significant difference from day 0, $p < 0.05$. Scale bars = 50 μm.

 © The Author(s)

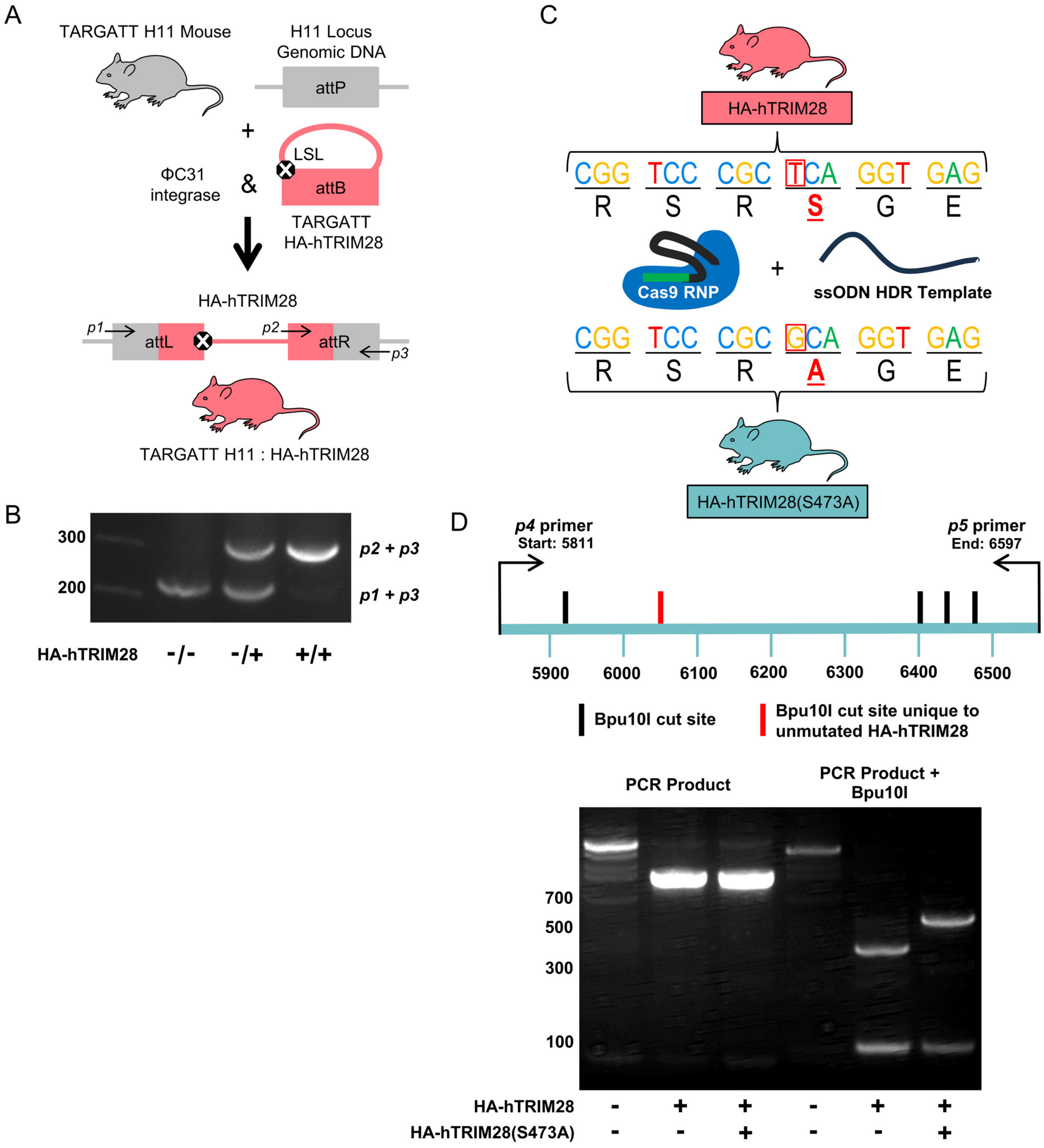

Figure EV4. Strategy for creating mice that allow for the tamoxifen-inducible expression of human TRIM28 or a S473A phosphodefective mutant of hTRIM28.

(**A**) Embryos from TARGATT mice that contained an attP integration site in the H11 locus were injected with ΦC31 integrase and an HA-hTRIM28 TARGATT vector that contained a LoxP-Stop-LoxP (LSL) cassette and an attB integration sequence. (**B**) As illustrated in A, three primers (*p1*, *p2*, and *p3*) were used to confirm the successful integration of the HA-hTRIM28 vector into the genomic DNA of the offspring. (**C**) CRISPR-Cas9-mediated homologous-directed repair was used to make a single point mutation in TARGATT H11 : HA-hTRIM28 mice that switched the serine 473 residue of hTRIM28 to a non-phosphorylatable alanine (HA-hTRIM28(S473A). (**D**) Primers *p4* and *p5* along with Bpu10I digestion were used to confirm the differences in the genomic DNA of the mice that expressed HA-hTRIM28 versus HA-hTRIM28(S473A).

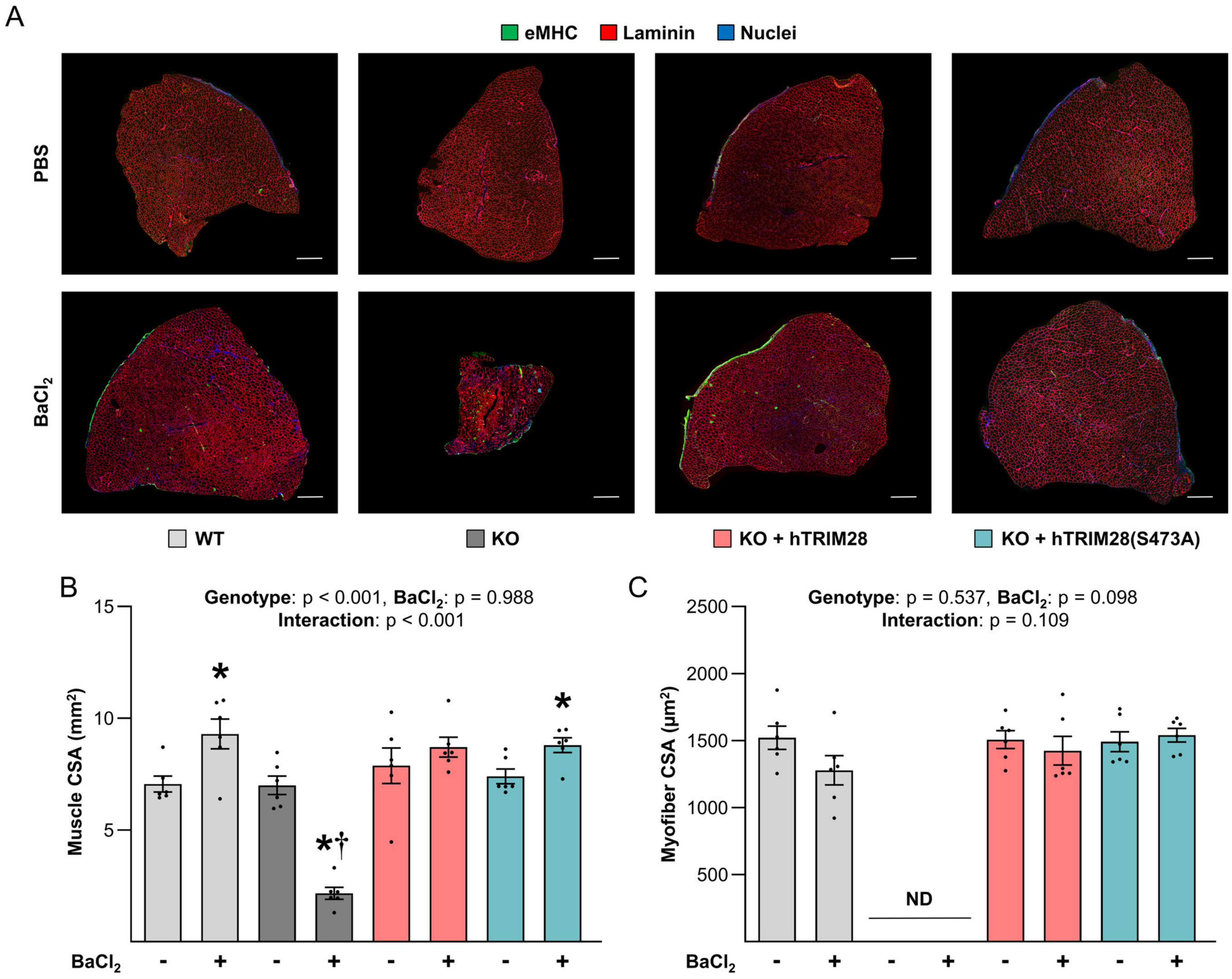

Figure EV5. TRIM28(S473) phosphorylation in satellite cells is not required for the restoration of muscle CSA or myofiber CSA following $BaCl_2$-induced injury.

At 14 days post tamoxifen the tibialis anterior muscles of wild-type (WT) mice, tamoxifen-inducible and satellite cell-specific TRIM28 knockout mice (KO) mice, as well as KO mice that contain tamoxifen-inducible "rescue" expression of hTRIM28 (KO + hTRIM28) or phosphodefective hTRIM28 (KO + hTRIM28(S473A)) were injected with $BaCl_2$ (+) to induce injury or PBS (−) as a control condition. The tibialis anterior muscles were collected after a 21-day recovery period. (**A**) Mid-belly cross sections of the muscles were subjected to immunohistochemistry for eMHC, laminin, and nuclei. Scale bar = 500 µm. (**B**) Measurements of whole muscle cross-sectional area (CSA) (two-way repeated-measures ANOVA, $n = 6$/group, *$p < 0.001$, $= 0.002$, $= 0.036$, †$p < 0.001$), and (**C**) the mean myofiber CSA per muscle (two-way repeated-measures ANOVA, $n = 6$/group). Values are presented as the group mean ± SEM, $n = 6$/group. Due to the absence of clear myofibers in the $BaCl_2$-treated muscles of KO mice, myofiber CSA data for these mice was not determined (ND). * indicates a significant effect of $BaCl_2$ within a given genotype, † indicates significant difference from the $BaCl_2$ treated WT condition, $p < 0.05$.

 © The Author(s)
